# Triple gene editing in porcine embryos using electroporation alone or in combination with microinjection

**DOI:** 10.14202/vetworld.2022.496-501

**Published:** 2022-02-27

**Authors:** Zhao Namula, Quynh Anh Le, Manita Wittayarat, Qingyi Lin, Koki Takebayashi, Maki Hirata, Lanh Thi Kim Do, Fuminori Tanihara, Takeshige Otoi

**Affiliations:** 1 Bio-Innovation Research Center, Tokushima University, 7793233 Tokushima, Japan; 2 Department of Veterinary Medicine, College of Coastal Agricultural Sciences, Guangdong Ocean University, 524088 Guangdong, China; 3 Laboratory of Animal Reproduction, Faculty of Bioscience and Bioindustry, Tokushima University, 7793233 Tokushima, Japan; 4 Faculty of Veterinary Science, Prince of Songkla University, 90110 Songkhla, Thailand; 5 Department of Animal Theriogenology and Surgery, Faculty of Veterinary Medicine, Vietnam National University of Agriculture, 100000 Hanoi, Vietnam; 6 Center for Development of Advanced Medical Technology, Jichi Medical University, 3290498 Tochigi, Japan

**Keywords:** clustered regularly interspaced short palindromic repeats/Cas9, electroporation, gene editing, microinjection, porcine zygotes

## Abstract

**Background and Aim:**

We previously developed the gene-editing by electroporation (EP) of Cas9 protein method, in which the CRISPR/Cas9 system was introduced into porcine *in vitro* fertilized (IVF) zygotes through EP to disrupt a target gene. This method should be further developed, and a combination of EP and MI methods should be evaluated in pigs. This study aimed to determine that a combination of microinjection (MI) and EP of CRISPR/Cas9 system could increase the rates of biallelic mutation for triple-gene knockout in porcine blastocysts. We targeted the pancreatic and duodenal homeobox1 (*PDX1*) gene using cytoplasmic MI 1 h before or after EP, which was used to edit alpha-1,3-galactosyltransferase (*GGTA1*) and cytidine 32 monophosphate-N-acetylneuraminic acid hydroxylase (*CMAH*) genes in porcine zygotes.

**Materials and Methods:**

We introduced guide RNAs targeting *PDX1*, *GGTA1*, and *CMAH* with the Cas9 protein into IVF zygotes (one-cell stage) through EP 10 h after the start of IVF (IVF; EP group) or in combination with MI (1 h before, MI-EP group, or after EP treatment EP-MI group) and evaluated the blastocyst formation rate and efficiency of target mutations in the resulting blastocysts.

**Results:**

Our results revealed a significant reduction in the rate of blastocyst formation in the two groups that underwent MI before and after EP (MI-EP and EP-MI group), compared with that in the groups treated with EP alone (EP group) (p=0.0224 and p<0.0001, respectively) and control (p=0.0029 and p<0.0001, respectively). There was no significant difference in the total mutation rates among the treatment groups in the resulting blastocysts. As an only positive effect of additional MI treatment, the rate of blastocysts carrying biallelic mutations in at least one target gene was higher in the MI-EP group than in the EP group. However, there was no difference in the rates of embryos carrying biallelic mutations in more than 2 target genes.

**Conclusion:**

These results indicate that although a combination of MI and EP does not improve the mutation efficiency or biallelic mutation for triple-gene knockout, MI treatment before EP is better to reduce mortality in porcine zygotic gene editing through a combination of MI and EP.

## Introduction

Pancreatic diseases are one of the most challenging disorders as they cause high morbidity and mortality in humans [1]; hence, research related to pig-to-human pancreas xenotransplantation has become a focus over the past decade, particularly with respect to the production of a functioning pancreas from pluripotent cells, such as induced pluripotent stem cells (iPS cells) [2]. Blastocyst complementation can be used to generate human entire organs from iPS cells in genetically modified pigs. The pigs are allowed to grow to a mature age before transplantation of the organ back into human patients [3-5]. Hyperacute rejection has been considered an unavoidable consequence of the transplantation of vascularized xenograft organs between two phylogenetically distant species [6]. Alpha-1,3-galactosyltransferase (*GGTA1*) and cytidine-32-monophosphate-N-acetylneuraminic acid hydroxylase (*CMAH*) genes are the main xenoantigens associated with the hyperacute rejection of xenografts [7-9], and the pancreatic and duodenal homeobox1 (*PDX1*) gene is important for pancreatic development in the fetus [10-12]. Therefore, the production of *GGTA1/CMAH/PDX1*-triple-gene knockout pigs using the blastocyst complementary method is required for the process of developing a pig-to-human pancreas xenotransplantation model.

Genetically modified pigs are generally produced using somatic cell nuclear transfer. After the improvement of gene-editing technology, gene modification in embryos has been dramatically improved. The clustered regularly interspaced short palindromic repeats (CRISPR)-Cas9 (CRISPR/Cas9) system is a highly efficient gene editing method that is used to create genetically modified blastocysts through cytoplasmic microinjection (MI) and electroporation (EP) into zygotes [13-15]. The basis of our hypothesis is that a combination of MI and EP methods could increase the rates of biallelic mutations for triple-gene knockout in porcine blastocysts because each particular gene might prefer different delivery methods of gene editing machinery. If our hypothesis is correct, it will be possible to efficiently generate human-sized pancreases derived from pluripotent stem cells using xenogeneic antigen reduced pigs and provide an opportunity to develop future therapies using pig-to-human pancreas xenotransplantation.

We previously developed the gene-editing by EP of Cas9 protein method in which the CRISPR/Cas9 system (Cas9 protein and the guide RNA [gRNA]) was introduced into porcine *in vitro* fertilized (IVF) zygotes through EP to disrupt a target gene [15]. This method should be further developed, and a combination of EP and MI methods should be evaluated in pigs.

This study aimed to determine that a combination of MI and EP of CRISPR/Cas9 system could increase the rates of biallelic mutation for triple-gene knockout in porcine blastocysts. We targeted the *PDX1* gene using cytoplasmic MI before or after EP, which was used to edit *GGTA1* and *CMAH* genes in porcine zygotes. This study will provide a basis for further optimizing multigene editing in porcine embryos and might contribute to the efficient generation of gene-modified pigs.

## Materials and Methods

### Ethical approval

This study was approved by the Institutional Animal Care and Use Committee of Tokushima University (approval number: T28-21).

### Study period and location

This study was conducted from February 2021 to April 2021. All laboratory works were conducted at the Bio-Innovation Research Center of Tokushima University and Laboratory of Animal Reproduction, Faculty of Bioscience and Bioindustry, Tokushima University.

### Oocyte collection, in vitro maturation (IVM), and IVF

Oocyte collection, IVM, and IVF were performed as described previously [16]. Briefly, pig ovaries were obtained from prepubertal crossed gilts at a local slaughterhouse. Cumulus-oocyte complexes were collected and cultured in a maturation medium. After IVF, the putative zygotes were denuded and cultured in porcine zygote medium (PZM-5; Research Institute for the Functional Peptides Co., Yamagata, Japan) until MI and EP treatments.

### EP

EP was performed as described previously [17]. Briefly, zygotes were electroporated (five 1 ms pulses at 25 V) with nuclease-free duplex buffer (Integrated DNA Technologies, Coralville, IA, USA) containing 100 ng/μL of gRNA (Alt-R™ CRISPR crRNAs and tracrRNA; Integrated DNA Technologies) and 100 ng/μL of Cas9 protein (Takara Bio, Inc., Shiga, Japan). After EP, the embryos were cultured in PZM-5. On day 3 after fertilization (day 0), all cleaved embryos were subsequently cultured in porcine blastocyst medium (PBM; Research Institute for the Functional Peptides Co., Japan) for 4 days.

### Cytoplasmic MI

Cytoplasmic MI was performed as described previously [18]. Briefly, the CRISPR/Cas9 components were injected into zygotes in a 20 μL drop of PZM-5 covered with mineral oil. The duplex buffer containing 100 ng/μL of gRNA and 100 ng/μL of Cas9 protein was loaded into the injection pipette (Femtotips II, Eppendorf, Hamburg, Germany) and injected into the cytoplasm based on air pressure using a microinjector (FemtoJet 4i; Eppendorf). After MI, the embryos were cultured in PZM-5 and PBM, as described previously herein.

### Design of gRNAs

gRNA was designed using the CRISPR direct web tool (https://crispr.dbcls.jp/) [19]. We confirmed that 12 bases at the 3’ end of the designed gRNAs had no identical sequence in the pig genome, except for the targeting region of the gene, to minimize the possibility of off-target effects using the COSMID web tool (https://crispr.bme.gatech.edu/) [20]. We designed three types of gRNAs targeting *PDX1*, *GGTA1*, and *CMAH*. The target sequences of gRNAs are listed in Table-1.

**Table 1:** gRNA and primer sequences targeting three genes.

Target gene	Target sequence (5′-3′)	PAM	Strand	Forward primer (5′-3′)	Reverse primer (5′-3′)
*PDX*	TGGCGAGGAGCAGTACTACG	CGG	Sense	ATAGAAGTCCAAATATTTTCCCCGC	ACCTCGTACGGGGAGATGTC
*CMAH*	GAAGCTGCCAATCTCAAGGA	AGG	Sense	GCTGTCAATGCTCAGGGATT	TGCCAAACCTAATTGGGAGA
*GGTA1*	AGACGCTATAGGCAACGAAA	AGG	Sense	AAAAGGGGAGCACTGAACCT	CCTGTCGGGAATGTTCTCAT

*GGTA1*=Alpha-1,3-galactosyltransferase, CMAH=Cytidine-32-monophosphate-N-acetylneuraminic acid hydroxylase, *PDX*=Pancreatic and duodenal homeobo×1, gRNA=Guide RNA

### Analysis of targeted gene sequence after MI and EP

Analysis of the targeted gene sequence was performed as described previously [17]. Genomic DNA isolated from the resulting blastocysts was subjected to a polymerase chain reaction (PCR) using specific primers (Table-1). The PCR products were analyzed using Sanger sequencing and the tracking of indels by decomposition (TIDE) bioinformatics package to determine the genotype of each blastocyst, as described previously [21]. TIDE is a web tool (available at http://tide.nki.nl) designed to accurately quantify the editing efficacy and simultaneously identify the predominant types of indels in the targeted pool of cells [22]. This enabled us to determine genotypes of blastocysts from the resulting sequencing traces of Sanger sequencing analysis. Blastocysts that did not carry wild-type (WT) sequences were classified as having biallelic mutations. Blastocysts that carried more than 1 type of mutation, in addition to the WT sequence, were classified as mosaics. Blastocysts that carried only the WT sequence were classified as WT.

### Experimental design

To evaluate the effects of EP alone or in combination with MI for genome delivery on the blastocyst formation rate and the efficiency of target mutations in the resulting blastocysts, we introduced gRNAs targeting *PDX1*, *GGTA1*, and *CMAH* with the Cas9 protein into IVF zygotes (one-cell stage) through EI alone or in combination with MI. Zygotes were randomly assigned to three treatment groups. In one group, the three gRNAs and Cas9 protein were introduced into zygotes through EP 10 h after the start of IVF (EP group). In the other two groups, two gRNAs targeting *GGTA1* and *CMAH* were introduced into zygotes through EP 10 h after IVF. Then, gRNA targeting *PDX1* was microinjected into the zygotes 1 h before (MI-EP group) or after EP treatment (EP-MI group). As a control, some zygotes were cultured with PZM-5 and PBM for 7 days without EP or MI.

### Statistical analysis

All data were subjected to arcsine transformation before performing analysis of variance (ANOVA). The transformed data were tested using ANOVA, followed by Fisher’s protected least significant difference test, using StatView software (Abacus Concepts, Berkeley, CA, USA). The percentages of edited and biallelic blastocysts relative to the total number of blastocysts were analyzed using Chi-squared analysis with Yates’ correction. Differences were considered statistically significant when p≤0.05 was considered.

## Results

**Figure-1:**
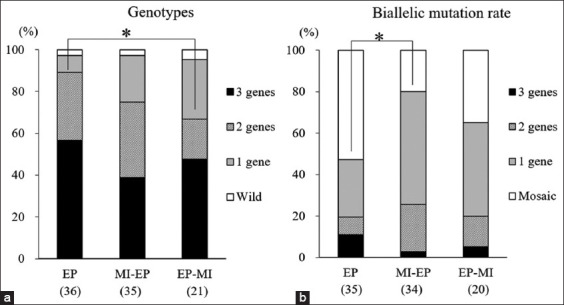
Introduction of the clustered regularly interspaced short palindromic repeats/Cas9 system targeting pancreatic and duodenal homeobox1 (*PDX1*), alpha-1,3-galactosyltransferase (*GGTA1*), and cytidine-32-monophosphate-Nacetylneuraminic acid hydroxylase (*CMAH)* genes into *in vitro* fertilized (IVF) zygotes through electroporation (EP) alone or in combination with microinjection (MI). (a) Genotypes of blastocysts. The proportions represent the percentages of blastocysts carrying the mutation number of each target gene in the total examined blastocysts. *The rate of blastocysts with mutations in at least two target genes was significantly higher in the EP group than in the EP-MI group. p<0.05. (b) Biallelic mutations in gene-edited blastocysts. The proportions represent the percentage of biallelic mutation events in the gene-edited blastocysts. *The rate of blastocysts carrying biallelic mutations in at least one target gene was significantly higher in the MI-EP group than in the EP group. p<0.05. EP: Three guide RNAs (gRNAs) targeting *PDX1*, *GGTA1*, and *CMAH* were introduced into zygotes through EP at 10 h after the start of IVF; MI-EP: gRNA targeting *PDX1* was microinjected into the zygotes 1 h before EP of two gRNAs targeting *GGTA1* and *CMAH*; EP-MI: gRNA targeting *PDX1* was microinjected into the zygotes 1 h after the EP of two gRNAs targeting *GGTA1* and *CMAH*. The numbers within parentheses on the X-axis indicate the total number of examined samples.

As shown in Table-2, the development rates of zygotes treated with a combination of EP and MI (MI-EP and EP-MI group) were significantly lower than those of zygotes treated with EP alone (EP group) (p<0.0001 and p<0.0001, respectively) and control zygotes (p<0.0001 and p<0.0001, respectively). Moreover, the blastocyst formation rate of zygotes with MI treatment after EP (EP-MI group) significantly decreased compared to those with MI treatment before EP (MI-EP group) (p=0.0045). The percentages of blastocysts carrying mutations in one, two, or three genes were 8.1%, 32.4%, and 56.8% in the EP group, 22.2%, 36.1%, and 38.9% in the MI-EP group, and 28.6%, 19.0%, and 47.6% in the EP-MI group, respectively (Figure-1a). The total mutation rates in blastocysts did not differ among the treatment groups (97.3%, 97.2%, and 95.2% in the EP, MI-EP, and EP-MI groups, respectively). Of the embryos examined, only one blastocyst carried the WT sequence in each treatment group. Moreover, the rate of blastocysts with mutations in at least two target genes was significantly higher (p=0.0355) in the EP group (89.2%) than in the EP-MI group (66.6%).

**Table 2:** Effects of EP alone or in combination with MI on the development of porcine zygotes*.

Experiment**	No. of embryos examined	No. (%) of embryos	

		Cleaved	Developed to blastocysts
Control	452	425 (94.0±0.9)^a^	117 (25.9±2.4)^a^
EP	468	439 (93.8±1.2)^a^	109 (23.4±2.1)^a^
MI-EP	454	302 (66.6±3.6)^b^	71 (15.7±2.7)^b^
EP-MI	466	220 (47.1±3.3)^c^	28 ( 6.0±1.5)^c^

* Nine replicate trials were performed. Percentages are expressed as the mean±SEM.

** As a control, zygotes were cultured without the EP and MI treatments. EP: Zygotes were electroporated 10 h after IVF. MI-EP: Zygotes were microinjected 9 h after IVF and then electroporated 1 h later. EP-MI: Zygotes were electroporated 10 h after IVF and microinjected 1 h later. ^a-c^ Values with different superscripts in the same column are significantly different (p<0.05). MI=Microinjection, EP=Electroporation, IVF=*In vitro* fertilized

The percentages of blastocysts carrying biallelic mutations in one, two, or three genes were 27.8%, 8.3%, and 11.1% in the EP group, 54.3%, 22.9%, and 2.9% in the MI-EP group, and 45.0%, 15.0%, and 5.0% in the EP-MI group, respectively (Figure-1b). Although the rate of blastocysts carrying biallelic mutations in at least one target gene was higher in the MI-EP group (80.0%) than in the EP group (47.2%), there was no difference in the rates of embryos carrying biallelic mutations in more than 2 target genes (19.4%, 25.8%, and 20.0% in the EP, MI-EP, and EP-MI groups, respectively; Figure-1b).

## Discussion

Application of the CRISPR/Cas9 strategy has the potential to rapidly increase the production of genetically engineered animals using either MI or EP into zygotes [13-15]. Cytoplasmic MI is a commonly used technique for delivering CRISPR/Cas9 components for targeted mutagenesis in organisms, and some studies have shown its efficacy in generating knockout mice [23,24]. However, since this technique requires high-level technical skills and causes a large percentage of mosaicism [25,26], we previously developed the EP protocol [17], which is described in the methods section of this study to overcome the obstacle of MI. Although we successfully used the CRISPR/Cas9 system with the EP technique to produce different types of mutated blastocysts with acceptable quality [17,21], we are still limited in generating biallelic mutations in multiple gene knockout targets. Surprisingly, our recent findings found that blastocysts with the highest rates of biallelic mutations and mutation efficiencies of certain genes were from 1-cell stage embryos edited using the MI method [27]. Thus, a combination of MI and EP methods might help to improve biallelic mutation rates for multigene knockout. In the present study, we compared the gene-editing efficiency and developmental rates of *GGTA1/CMAH/PDX1* triple-gene knockout in porcine blastocysts, based on gene editing machinery delivery.

Our results showed that the use of MI remarkably decreased the rates of embryonic cleavage, and embryos developed into blastocysts. A high concentration of injected Cas9 protein can cause a reduction in embryo development; however, the Cas9 protein (160 kDa) used in this study was at 100 ng/μL, the same concentration as that in our previous study, which was suggested to result in low toxicity [27]. This means that embryonic damage is probably due to another reason other than Cas9 protein toxicity. MI can damage the embryos as it normally perforates the cell membrane before sufficient material from the micropipette is introduced into the embryos using high pressure during short periods of time [28]. High embryo lethality due to the vulnerability of the pronuclei has been reported when MI was used to transfer CRISPR/Cas9 components [29]. Although in the present study, we used cytoplasmic MI to produce triple-gene knockout porcine blastocysts, which might result in decreased embryonic damage compared to that in the pronucleus MI, it is still possible that accidental injection into the pronuclei structure might occur due to the lipid granules in the cytoplasm that can interfere with pronuclei visualization in porcine zygotes [30]. The group that underwent cytoplasmic MI after EP (MI was conducted approximately 11 h after IVF) had a significantly lower rate of embryo development. At that time, pronucleus structures inside the zygotes could be present since porcine pronuclei form 8-9 h after the injection of sperm [31]. To improve cytoplasmic MI, changing the timing of MI to be slightly earlier than 8-9 h after IVF could be the key to reducing embryo mortality. However, further studies are needed to confirm this and determine its editing efficiency.

This study lacked an experimental group that introduced three gRNAs and Cas9 protein into zygotes through MI 10 h after the start of IVF. In a previous study, we compared the rates of blastocyst formation and mutation in the resulting blastocysts from zygotes between MI and EP treatment methods [27]. Although the mutation rates of blastocysts were statistically comparable between both groups, the blastocyst formation rate tended to decrease in the MI treatment group compared with that in the EP treatment group. Therefore, the simultaneous introduction of three gRNAs was only performed using EP. However, this is one of the limitations of our study, and we should further evaluate multiple gene editing efficiency through different types of delivery.

The percentages of edited and biallelic blastocysts among the total number of blastocysts were similar in all groups. Moreover, the rate of blastocysts with mutations in at least two target genes was higher in the EP group than in the EP-MI group. Cytoplasmic MI requires a certain gene structure and a high number of gene-editing components. Although we previously found that increasing the concentration of Cas9 protein and gRNA from 20 to 100 ng/μL could efficiently generate blastocysts with biallelic mutants, blastocysts with mosaicism still exist [18]. Some genes were targeted for mutagenesis with higher concentrations of gRNA injection (200 ng/μL) [32,33], suggesting that the gRNA concentration might be an essential factor to improve the mutation efficiency when MI is used.

## Conclusion

In this study, we demonstrated that a combination of MI and EP did not increase the rates of mutation efficiency or biallelic mutation in *GGTA1/CMAH/PDX1* triple-gene knockout porcine blastocysts. Moreover, MI was found to have a negative effect on embryo developmental rates. Factors that might affect the results of the current study, such as the timing of cytoplasmic MI and the suitable concentration of gRNA used, should be evaluated and optimized in a further study to improve the efficacy of a combination of MI and EP for delivery of the CRISPR/Cas9 system into zygotes and to reduce embryo toxicity.

## Authors’ Contributions

ZN, FT, and TO: Conceived the study. ZN: Performed the majority of experiments and wrote the manuscript. TO: Designed the study and coordinated the experiments. FT and TO: Reviewed the manuscript. MH: Performed the sequencing analysis. QAL, QL, KT, and LTKD: Contributed to the laboratory work and statistical analysis. MW: Revised the manuscript. All authors read and approved the final manuscript.
